# Sleep Efficiency and Sleep Onset Latency in One Saskatchewan First Nation

**DOI:** 10.3390/clockssleep6010004

**Published:** 2024-01-10

**Authors:** Chandima P. Karunanayake, Punam Pahwa, Shelley Kirychuk, Mark Fenton, Vivian R. Ramsden, Jeremy Seeseequasis, Warren Seesequasis, Robert Skomro, Donna C. Rennie, Kathleen McMullin, Brooke P. Russell, Niels Koehncke, Sylvia Abonyi, Malcolm King, James A. Dosman

**Affiliations:** 1 Canadian Centre for Rural and Agricultural Health, University of Saskatchewan, 104 Clinic Place, Saskatoon, SK S7N 2Z4, Canada; pup165@mail.usask.ca (P.P.); shelley.kirychuk@usask.ca (S.K.); jts124@mail.usask.ca (J.S.); kathleen.mcmullin@usask.ca (K.M.); bpr053@mail.usask.ca (B.P.R.); niels.koehncke@usask.ca (N.K.); james.dosman@usask.ca (J.A.D.); 2 Department of Community Health & Epidemiology, College of Medicine, University of Saskatchewan, 107 Wiggins Road, Saskatoon, SK S7N 5E5, Canada; sylvia.abonyi@usask.ca (S.A.); malcolm.king@usask.ca (M.K.); 3 Department of Medicine, University of Saskatchewan, Royal University Hospital, 103 Hospital Drive, Saskatoon, SK S7N 0W8, Canada; mef132@mail.usask.ca (M.F.); r.skomro@usask.ca (R.S.); 4 Department of Academic Family Medicine, University of Saskatchewan, West Winds Primary Health Centre, 3311 Fairlight Drive, Saskatoon, SK S7M 3Y5, Canada; viv.ramsden@usask.ca; 5 Community A, P.O. Box 96, Duck Lake, SK S0K 1J0, Canada; wseesequasis@beardysband.com; 6 College of Nursing, University of Saskatchewan, 104 Clinic Place, Saskatoon, SK S7N 2Z4, Canada; donna.rennie@usask.ca

**Keywords:** sleep efficiency, sleep onset latency, First Nations, poor sleep, chronic pain, anxiety, smoking

## Abstract

Background: Sleep efficiency and sleep onset latency are two measures that can be used to assess sleep quality. Factors that are related to sleep quality include age, sex, sociodemographic factors, and physical and mental health status. This study examines factors related to sleep efficiency and sleep onset latency in one First Nation in Saskatchewan, Canada. Methods: A baseline survey of the First Nations Sleep Health project was completed between 2018 and 2019 in collaboration with two Cree First Nations. One-night actigraphy evaluations were completed within one of the two First Nations. Objective actigraphy evaluations included sleep efficiency and sleep onset latency. A total of 167 individuals participated, and of these, 156 observations were available for analysis. Statistical analysis was conducted using logistic and linear regression models. Results: More females (61%) than males participated in the actigraphy study, with the mean age being higher for females (39.6 years) than males (35.0 years). The mean sleep efficiency was 83.38%, and the mean sleep onset latency was 20.74 (SD = 27.25) minutes. Age, chronic pain, ever having high blood pressure, and smoking inside the house were associated with an increased risk of poor sleep efficiency in the multiple logistic regression model. Age, chronic pain, ever having anxiety, heart-related illness, and smoking inside the house were associated with longer sleep onset latency in the multiple linear regression model. Conclusions: Sleep efficiency and sleep onset latency were associated with physical and environmental factors in this First Nation.

## 1. Introduction

Sleep efficiency, the amount of time actually spent sleeping while in bed as a percentage, and sleep onset latency, the length of time it takes to fall asleep, are two measures that can be used to assess sleep quality [1]. Other measures are sleep waking (how many times the person wakes during the night) and wakefulness (how many minutes are spent awake during the night after sleep onset) [1]. Many studies have shown that factors related to sleep quality include age [2,3], sex [2,4], sociodemographic factors [5,6,7,8,9], smoking [10,11], health conditions including hypertension [3,4,12,13,14,15,16,17,18,19,20,21], cardiovascular disease [4,22,23,24,25,26], diabetes [27], heart failure [28], mental health issues including cognitive impairment and Alzheimer’s disease [29], anxiety [30,31,32,33,34], and depression [3]. Other sleep-related variables such as pain [35,36,37,38,39], bad dreams [2,40], nocturia [2], environmental noise [41], and secondhand smoke [42,43,44,45] were also related to sleep quality. Sleep medications significantly increase total sleep time and sleep efficiency and decrease sleep onset latency [46]. In addition, physical activity [47] and energy expenditure [48] are also associated with sleep efficiency. It is also known that psychological disorders [49,50], cardiovascular disease [51,52], and metabolic effects [53,54] are associated with shorter sleep duration.

Recent findings from the 2022 Canadian Community Health Survey reported that among adults aged 18 to 64, 61% reported high sleep quality, compared with 71% among adults aged 65 and older [55]. However, this survey excluded people living on First Nations reserves and other Indigenous settlements. Therefore, little is known about sleep quality, including sleep efficiency and sleep onset latency, among First Nations peoples in Canada. Also, there are no studies reported in the literature about risk factors and their possible associations with sleep efficiency and sleep onset latency in First Nations peoples. To bridge the gap in this current research, the objective of this study was to examine the factors related to sleep efficiency and sleep onset latency in one First Nation in Saskatchewan, Canada.

## 2. Results

In this one-night actigraphy study, more females (61%) participated than males. The mean age is higher for females than the males who participated (Table 1). The total sleep time was low (<6 h) during this one-night study. The mean sleep efficiency was 83.38%, and the mean sleep onset latency was 20.74 (SD = 27.25) minutes (Table 2) from the one-night actigraphy data. Sleep efficiency (%) data were normally distributed (the Kolmogorov–Smirnov test was used to test for normality with the resulting statistics = 0.063; df = 156; *p* = 0.200). The median of one-night actigraphy measured sleep efficiency was 83.92%. According to the definition, 55.1% (86/156) had poor sleep efficiency, and 44.9% (70/156) had good sleep efficiency. With the same cutoffs for self-reported sleep efficiency, 7.8% (12/153) had poor sleep efficiency. Sleep efficiency by objective (actigraphy) measurement and the self-reported subjective (using PSQI) measurement of sleep efficiency were both available for 153 participants. Sleep efficiency objective (actigraphy) measurements (83.33%; SD = 7.91%) were significantly different from self-reported subjective (using PSQI) measurements (94.19%; SD = 6.13%) of sleep efficiency (Table 2). The *p*-value was <0.0001. A paired-t test analysis revealed that log-transformed 126 sleep onset latency self-reported measurements (1.27; SD = 0.43) were not significantly different from sleep onset latency objective measurements (1.21; SD = 0.43) by actigraphy (*p*-value was 0.245).

Univariable analysis showed that ever high blood pressure and smoking inside the home were significantly associated (*p* < 0.05) with an increased risk of poor sleep efficiency (Table 3). In addition, education, smoking status, chronic pain, having ever had a respiratory illness, a moldy or musty smell, and signs of mold in any living area served as candidate variables (*p* < 0.25) for multivariable analysis. Age, sex, taking prescription medication on a regular basis, and drinking caffeinated beverages within two hours of going to bed were candidate variables for the multivariable regression model because of their clinical importance. The multivariable logistic regression model showed that age, chronic pain, ever having high blood pressure, and smoking inside the home were associated with an increased risk of poor sleep efficiency (Table 4).

Table 5 shows the results of univariable and multivariable linear regression analyses of log-transformed sleep onset latency (N = 126). Regression coefficients transformed to the original scale are shown in Table 5 within brackets. The coefficient of determination (R^2^) of the multivariable regression model is 0.206. Longer sleep onset latency was associated with the participant’s age, exposure to smoke inside the home, chronic pain, and those who have been diagnosed with anxiety or a heart-related illness.

## 3. Discussion

In the current study, we explored the prevalence of sleep efficiency, sleep onset latency, and associated risk factors in one First Nation. The main observations were that the mean sleep efficiency was somewhat lower than the reference range (85.0%) [56], and the mean sleep onset latency was somewhat greater than the reference range (10–20 min) [57]. The prevalence of sleep efficiency of ≤85% was 55.1%. The prevalence of sleep onset latency >20 min was 34.0%. Among Canadian adults aged 18 years and older, the mean sleep duration was 8.0 h per night, with 72.7% meeting sleep duration recommendations [49]. In the current study, sleep duration was lower (5.8 h) than Canadian norms. Shorter sleep duration could lead to many health problems, such as psychological and mental disorders [49,50], cardiovascular diseases [51,52], and issues with metabolic systems [53,54]. Therefore, adequate sleep duration may be important for preventing these health problems. Factors associated with poor sleep efficiency were age, chronic pain, high blood pressure, and exposure to smoke inside the home. Factors associated with longer sleep onset latency were age, exposure to smoke inside the home, chronic pain, and being diagnosed with anxiety and heart-related illnesses.

One of the factors associated with sleep efficiency and sleep onset latency is age. Studies have shown that sleep quality changes with age [2,3]. Desjardins et al. [2] reported that poor sleep efficiency was prevalent among older people. Another cross-sectional study by Didikoalu et al. [3] reported that with the increase in age, there was a decrease in sleep efficiency. In adults, sleep onset latency increases gradually with age [58]. In contrast to these studies, the current study found a higher risk of poor sleep efficiency among the 18- to 54-year-old age group compared to the 55-year-old and older age groups. Similar to poor sleep efficiency, a higher risk of longer sleep onset latency was associated with the 18- to 54-year-old age group.

Studies have shown a relationship between sleep quality and chronic pain [35,36,37,38,39]. The results from a cross-sectional study showed a positive direct effect of chronic pain on poor sleep quality and poor sleep efficiency [35]. Abeler et al. [36] reported that participants with chronic musculoskeletal pain exhibited more subjective sleep disturbance and moderately worse sleep efficiency compared to pain-free controls. There was a close interaction between central sensitization and sleep disturbances in people with chronic pain [38]. These researchers have also shown that this interaction was bidirectional. Smith et al. [59] reported that sleep quality and pain intensity were related, and sleep quality was related to the patient’s mood and physical function factors [39]. Similar to sleep efficiency, there was a link between sleep onset latency and the intensity of chronic pain [37]. This study reported a 3.5-fold higher risk of poor sleep efficiency in people with chronic pain. Further, people with chronic pain have longer sleep onset latency.

Canadian First Nation peoples living on-reserve have higher rates of non-traditional use of tobacco than those living off-reserve [60,61]. Aboriginal peoples were also twice as likely to be exposed to second-hand smoke in the home [62]. Studies have shown that smoke exposure is related to sleep quality [42,43,44,45]. A study of never-smokers revealed that exposure to secondhand smoke, frequency, and duration of exposure were associated with poor sleep quality and sleep onset latency in never-smoking adults overall [43]. The authors also reported a dose-response (frequency and duration of exposure) relationship between secondhand smoke exposure and poor sleep quality and sleep onset latency among women but not in men [43]. Zandy et al. [42] reported that secondhand smoke exposure is associated with sleep duration, trouble falling and staying asleep, and sleep dissatisfaction. Another study by Valentino et al. [44] reported that young adults who self-reported higher levels of secondhand smoke exposure were also more likely to report sleep disturbances and lower sleep quality. Toyama et al. [45] reported that associations were found between secondhand smoke exposure and both poor sleep quality and sleep bruxism. This study further confirmed the available evidence. There was a 2.9-fold higher risk of poor sleep efficiency in people exposed to secondhand smoke. Further, people exposed to secondhand smoke have longer sleep onset latency.

Studies have shown an association between hypertension and sleep efficiency [3,4,12,13,15,17,18]. Yuan and others [12] reported that in a population of young and middle-aged populations, habitual sleep efficiency in the <65–75% group had an increased risk of developing hypertension compared to their counterparts. Another study reported that increased sleep efficiency was independently associated with lower systolic blood pressure [13]. A cross-sectional study of Japanese adults showed that reduced sleep efficiency was significantly associated with an increased prevalence of hypertension [15]. A study of young adolescents reported that higher sleep efficiency was associated with lower metabolic risk scores [17]. Another study of a healthy old age cohort revealed that sleep efficiency decreased with increasing age. Also, compared with low sleep efficiency, belonging to a high sleep efficiency group was associated with having a lower prevalence of hypertension and circulatory problems [3]. In another study, sleep efficiency was associated with higher systolic blood pressure [18]. This current study supports the evidence and reports that poor sleep efficiency is associated with high blood pressure.

This study reported that having a heart problem or atrial fibrillation was associated with sleep onset latency. Not many studies support this observation. One study of rural older adults in China revealed that poor sleep quality, including prolonged sleep onset latency and reduced sleep efficiency, was significantly associated with female sex and clinical co-morbidities such as hypertension, coronary heart disease, and chronic obstructive pulmonary disease [4]. In another study, clinical heart failure predicted a reduction in sleep quality, as indicated by sleep onset latency and sleep quality components of the Pittsburgh Sleep Quality Index [28].

Studies have shown an association between anxiety disorders and sleep disturbances. Patients with panic disorder reported longer sleep onset latency, increased time awake, and reduced sleep efficiency [31,33,34]. Sleep onset latency changes were associated with anxiety [30]. A study by Farris and others [32] indicated that anxiety sensitivity was significantly correlated with disturbances in sleep duration, subjective sleep quality, and sleep onset latency. This study also showed a positive correlation between longer sleep onset latency and whether the individual was ever diagnosed with anxiety.

### Strengths and Limitations

The strengths of this study included the fairly large number of participants and the inclusion of a number of potential factors, including lifestyle, sociodemographic, and sleep characteristics. This study is the first to our knowledge to examine sleep efficiency and sleep onset latency in adults living in a rural Cree First Nation in Saskatchewan, Canada, using actigraphy. However, one of the limitations is that the actigraphy was limited to one night. It has been recommended that at least five nights be spent assessing a reliable measure of sleep efficiency [63]. There were a few other concerns, such as not wearing the equipment during the entire night and the need to repeat the test if the information was not recorded. The average number of awakes was higher in this population compared to the general population, which needs further investigation.

The questionnaire survey data were self-reported, with possible recall bias. The willingness to participate in an actigraphy test was 40% (167/418) of those who completed the survey questionnaire. However, the participants in this sample volunteered for follow-up. Therefore, this is not a random sample of the population. An additional consideration in this study was that the population was young [mean age 35 years (SD = ±14.6 years) for males and 39.6 years (SD = ±14.9 years) for females]. Although associations of several factors were observed with sleep efficiency and sleep onset latency, causal relations could not be assessed due to the cross-sectional nature of the data.

## 4. Materials and Methods

### 4.1. Study Sample

The baseline survey of the First Nations Sleep Health Project (FNSHP) was completed between 2018 and 2019 in collaboration with the two Cree First Nations (Community A and Community B) in Saskatchewan, Canada. The methods were published elsewhere [64,65,66] and are only briefly described here. The overall goal of the FNSHP was to study the relationships between sleep disorders, risk factors, and co-morbidities and to evaluate local diagnosis and treatment. A Certificate of Approval was obtained from the University of Saskatchewan’s Biomedical Research Ethics Board (Certificate No. Bio #18-110). In addition, adherence to Chapter 9 (Research Involving the First Nations, Inuit, and Metis Peoples of Canada) in the Tri-Council Policy Statement: Ethical Conduct for Research Involving Humans was undertaken [67]. Participants provided written consent prior to engaging in this research project.

### 4.2. Data Collection

Research assistants were hired from the communities and trained to conduct the baseline surveys in their respective communities. Data were collected via interviewer-administered questionnaires (in Community A and B) and clinical assessments (only conducted in Community A). The survey collected information on demographic variables and individual and contextual determinants of sleep health. Objective clinical measurements included a Level 3 home overnight sleep test and actigraphy. This manuscript is based on data from the questionnaires and one-night home actigraphy collected in Community A. 

#### 4.2.1. Clinical Measurements

Anthropometric measurements (abdominal girth, neck circumference, hip circumference, height, and weight) were obtained. Height was measured against a wall using a fixed tape measure, with participants standing in socks on a hard floor. Weight was measured using a calibrated spring scale with participants in socks and dressed in indoor clothing. Using clinical measures of weight and height, body mass index (BMI) was calculated based on the equation BMI = weight (in kilograms)/(height (in meters))^2^ [68]. 

Trained research assistants prepared the actigraphy device (Phillips Respironics Actiwatch (AW) Spectrum Plus wristband device: ©Philips Respironics, Bend, OR, USA) before positioning the device on the left or right wrist. Once the Actiwatch was returned after wearing it for at least one night, the research assistants downloaded the results and checked to see if the test had been properly recorded. If yes, the participant was given an honorarium for completing the survey questionnaire and a one-night home test. If not, the participant was invited to redo the test. They were given an honorarium regardless of the successful completion of a test.

Objective sleep measurements (actigraphy) provide the bedtime, get-up time, time in bed (hours), total sleep time (hours), sleep onset latency (minutes), sleep efficiency (%), wake time after sleep onset (WASO) (minutes), and number of awakenings. Self-reported sleep measurements and the Pittsburgh Sleep Quality Index (PSQI) [69] were used to measure seven individual components: subjective sleep quality, sleep efficiency, sleep onset latency, sleep duration, sleep disturbances, use of sleep medication, and daytime dysfunction over the last month. This paper only considered two components: sleep efficiency and sleep onset latency.

Of the 233 persons who participated in a one-night sleep study, 168 participants completed one night of sleep actigraphy during July–December 2018 and May–August 2019. Examining the readings from the actigraphy, 12 minimal sleep periods of less than 180 min (3 h) were excluded from the analysis [70]. One hundred and fifty-six observations were available for analysis. Of those, self-reported sleep quality was available for 153 participants. For both log-transformed sleep onset latency and self-reported sleep onset latency, 126 observations were available for the analysis. 

#### 4.2.2. Definitions

Sleep efficiency from the Pittsburgh Sleep Quality Index (PSQI) and actigraphy was defined as sleep efficiency = (total # hours asleep)/(total # of hours in bed) × 100. For logistic regression modeling, cutoffs of >85% as good sleep efficiency and ≤85% as poor sleep efficiency were used [69]. Sleep onset latency, was defined as the time it takes a person to fall asleep after turning off the lights [1]. 

#### 4.2.3. Statistical Analysis

Statistical analyses were conducted using SPSS version 28 [IBM Corp. Released 2022. IBM SPSS Statistics for Windows, Version 28.0. Armonk, NY, USA: IBM Corp.]. Descriptive statistics, mean, median, and standard deviation (SD) were reported for continuous variables, and the *p*-value of the paired *t*-test was reported when comparing the means of paired samples. For categorical variables, frequency and percentages (%) were reported.

We used data obtained from actigraphy rather than self-reported data to determine the association between outcomes of sleep efficiency and sleep onset latency with independent variables of interest. Chi-squared tests were used to determine the univariable association of sleep efficiency prevalence with independent variables of interest. Multivariable logistic regression models were used to predict the relationship between a binary outcome of sleep efficiency (good or poor) and a set of explanatory variables. A series of logistic regression models were fitted to determine whether potential risk factors, confounders, and interactive effects contributed significantly to the prediction of sleep efficiency. 

Sleep onset latency was transformed by a common log transformation to normalize sleep onset latency data. Test of normality: the Kolmogorov–Smirnov statistic is 0.068 (df = 126) with a *p*-value of 0.200. Log-transformed sleep onset latency (>0 values) was used in the statistical analyses, and 27 observations with zero were not included in the analysis. A series of univariable linear regression models were run to identify the candidate variables for the multivariable linear regression model. Multivariable linear regression was used to analyze the relationship between the dependent variable of log-transformed sleep onset latency and the independent variables of interest. Based on the univariable analysis, variables with *p* < 0.20 and less than 25% missing information were candidates for the multivariable linear regression model. All statistically significant variables (*p* < 0.05), as well as important clinical factors (age, sex, chronic pain, use of prescription medication), were retained in the final multivariable regression model. Interactions between potential effect modifiers were examined and were retained in the final model if the *p*-value was <0.05.

## 5. Conclusions

Sleep efficiency and sleep onset latency were associated with physical and environmental factors in one First Nation. In this population, sleep efficiency was lower than the reference range, and sleep onset latency was greater than the reference range. Many reasons could be attributed to poor sleep, including a sleep disorder, a poor sleep environment, or another health condition. This study could suggest areas for further research to understand the sleep patterns among First Nation peoples.

## Figures and Tables

**Table 1:** Demographics of the study population.

	*n* (%)	Mean Age	Standard Deviation of Age	Minimum Age	Maximum Age
All	156 (100)	37.8	14.9	18	76
Male	61 (39.1)	35.0	14.6	18	76
Females	95 (60.9)	39.6	14.9	18	69

**Table 2:** Results from mean objective sleep measurements (actigraphy) and mean subjective measurements (using PSQI).

	One-Night Actigraphy Data (*n* = 156)		Self-Reported PSQI Scale Data (*n* = 153)	
	Mean ± SD	Median (Interquartile Range)	Mean ± SD	Median (Interquartile Range)
Total time in bed, in hours	6.91 ± 1.70	6.68 (2.49)	8.39 ± 2.16	8.00 (3.00)
Total sleep time, in hours	5.78 ± 1.56	5.78 (2.13)	7.94 ± 2.19	7.92 (2.83)
Sleep onset latency, in minutes	20. 74 ± 27.25	14.00 (23.50)	26.89 ± 26.86	20.00 (20.00)
Sleep efficiency %	83.38 ± 7.84	83.92 (10.88)	94.19 ± 6.13	96.43 (5.12)
Wake time after sleep onset (WASO), in minutes	38.06 ± 20.23	35.25 (21.38)	-	-
Number of awakes	27.76 ± 12.41	26.00 (15.00)	-	-

**Table 3:** Univariable associations between factors and sleep efficiency (poor vs. good) (*n* = 156).

Variables	Total *n* (%)	Sleep Efficiency *n* (%)		*p*-Value ^†^	OR (95% CI)	*p*-Value ^‡^
		Good (>85%)	Poor (≤85%)			
Sex						
Male	61 (39.1)	25 (35.7)	36 (41.9)	0.434	1.30 (0.68, 2.48)	0.434
Female	95 (60.9)	45 (64.3)	50 (58.1)		1.00	
Age groups						
18–54 years	127(81.4)	55 (78.6)	72 (83.7)	0.411	1.40 (0.62, 3.15)	0.412
55 years and over	29 (3.8)	15 (21.4)	14 (16.3)		1.00	
Body Mass Index						
Obese	74 (47.4)	33 (47.1)	41 (47.7)	0.659	1.24 (0.58, 2.65)	0.575
Overweight	40 (25.6)	16 (22.9)	24 (27.9)		1.50 (0.62, 3.60)	0.364
Neither overweight nor obese	42 (26.9)	21 (30.0)	21 (24.4)		1.00	
Education						
High school not completed	103 (66.5)	50 (71.4)	53 (62.4)	0.234	0.66 (0.34, 1.31)	0.235
High school or above	52 (33.5)	20 (28.6)	32 (37.6)		1.00	
Money left over at the end of the month						
Not enough money	91 (59.5)	39 (57.4)	52 (61.2)	0.627	1.41 (0.65, 3.09)	0.388
Just enough money	27 (17.6)	11 (16.2)	16 (18.8)		1.54 (0.56, 4.25)	0.404
Some money (ref)	35 (22.9)	18 (26.5)	17 (20.0)		1.00	
Drink caffeinated beverages within two hours of going to bed						
Yes	59 (38.6)	23 (33.8)	36 (42.4)	0.281	1.44 (0.74, 2.78)	0.282
No	94 (61.4)	45 (66.2)	49 (57.6)		1.00	
Smoking status						
Current smoker	113 (72.4)	48 (68.6)	65 (75.6)	0.345	2.03 (0.77, 5.35)	0.152
Ex-smoker	23 (14.7)	10 (14.3)	13 (15.1)		1.95 (0.58, 6.58)	0.282
Never smoker	20 (12.8)	12 (17.1)	8 (9.3)		1.00	
Physical activity at least 3 days per week						
Yes	76 (50.3)	34 (50.7)	42 (50.0)	0.927	0.97 (0.51, 1.85)	0.927
No	75 (49.7)	33 (49.3)	42 (50.0)		1.00	
Taking any prescription medication on a regular basis						
Yes	70 (45.2)	35 (50.0)	35 (41.2)	0.272	0.70 (0.37, 1.32)	0.273
No	85 (54.8)	35 (50.0)	50 (58.8)		1.00	
Sleep medication						
Yes	20 (13.0)	8 (11.6)	12 (14.1)	0.643	1.25 (0.48, 3.26)	0.644
No	134 (87.0)	61 (88.4)	73 (85.9)		1.00	
Traditional medication used for sleep *						
Yes	4 (2.6)	1 (1.4)	3 (3.5)	0.386	2.52 (0.26, 24.82)	0.427
No	151 (97.4)	69 (98.6)	82 (96.5)		1.00	
Ever had anxiety						
Yes	49 (35.0)	22 (34.9)	27 (35.1)	0.986	1.01 (0.50, 2.02)	0.986
No	91 (65.0)	41 (65.1)	50 (64.9)		1.00	
Chronic pain						
Yes	44 (30.1)	16 (23.5)	28 (35.9)	0.104	1.82 (0.88, 3.76)	0.106
No	102 (69.9)	52 (76.5)	50 (64.1)		1.00	
Ever had high blood pressure						
Yes	36 (27.1)	11 (17.7)	25 (35.2)	0.024	2.52 (1.12, 5.68)	0.026
No	97 (72.9)	51 (82.3)	46 (64.8)		1.00	
Heart-related illness						
Yes	17 (10.9)	9 (12.9)	8 (9.3)	0.479	0.69 (0.25, 1.91)	0.480
No	139 (89.1)	61 (87.1)	78 (90.7)		1.00	
Ever had a stroke *						
Yes	2 (1.3)	1 (1.5)	1 (1.2)	0.700	0.82 (0.05, 13.31)	0.887
No	149 (98.7)	67 (98.5)	82 (98.8)		1.00	
Ever had high cholesterol						
Yes	18 (12.7)	8 (11.9)	10 (13.3)	0.803	1.13 (0.42, 3.07)	0.803
No	124 (87.3)	59 (88.1)	65 (86.7)		1.00	
Ever had diabetes						
Yes	25 (17.5)	10 (15.6)	15 (19.0)	0.599	1.27 (0.53, 3.05)	0.599
No	118 (82.5)	54 (84.4)	64 (81.0)		1.00	
Respiratory illness (COPD/EMP/Asthma/CB/PNEU)						
Yes	39 (25.0)	14 (20.0)	25 (29.1)	0.193	0.61 (0.29, 1.29)	0.195
No	117 (75.0)	56 (80.0)	61 (70.9)		1.00	
Ever had reflux/heartburn						
Yes	54 (36.2)	22 (32.8)	32 (39.0)	0.434	1.31 (0.67, 2.57)	0.435
No	95 (63.8)	45 (67.2)	50 (61.0)		1.00	
Depression						
Yes	45 (31.7)	20 (32.3)	25 (31.3)	0.898	0.95 (0.47, 1.95)	0.898
No	97 (68.3)	42 (67.7)	55 (68.8)		1.00	
Ever sinus problems						
Yes	16 (10.8)	6 (9.0)	10 (12.3)	0.508	1.43 (0.49, 4.17)	0.510
No	132 (89.2)	61 (91.0)	71 (87.7)		1.00	
Sleep Apnea *						
Yes	6 (4.5)	1 (1.6)	5 (6.9)	0.143	4.55 (0.52, 40.06)	0.172
No	128 (95.5)	61 (98.4)	67 (93.1)		1.00	
Ever had post-traumatic stress disorder						
Yes	18 (12.6)	7 (11.1)	11 (13.8)	0.417	1.27 (0.46, 3.51)	0.637
No	125 (87.4)	56 (88.9)	69 (86.3)		1.00	
Wake up during the night due to terrifying dreams or nightmares or flashbacks to a traumatic event						
Yes	71 (45.5)	34 (48.6)	37 (43.0)	0.489	0.80 (0.42, 1.51)	0.489
No	85 (54.5)	36 (51.4)	49 (57.0)		1.00	
Wake up and get out of bed during the night						
Never	26 (16.8)	12 (17.1)	14 (16.5)	0.485	1.00	
1–2 times	91 (58.7)	44 (62.9)	47 (55.3)		0.92 (0.38, 2.19)	0.843
3 or more times	38 (24.5)	14 (20.0)	24 (28.2)		1.46 (0.53, 4.05)	0.457
Housing Conditions						
Crowding						
≤2 people/bedroom	114 (73.1)	53 (75.7)	61 (70.9)	0.503	1.00	
>2 people/bedroom	42 (26.9)	17 (24.3)	25 (29.1)		1.28 (0.62, 2.62)	0.503
Damage caused by dampness						
Yes	85 (55.2)	36 (52.9)	49 (57.0)	0.617	1.18 (0.62, 2.23)	0.617
No	69 (44.8)	32 (47.1)	37 (43.0)		1.00	
Moldy or musty smell						
Yes	82 (52.9)	41 (59.4)	41 (47.7)	0.145	0.62 (0.33, 1.18)	0.146
No	73 (47.1)	28 (40.6)	45 (52.3)		1.00	
Signs of mold in any living areas in your house						
Yes	81 (52.9)	32 (46.4)	49 (58.3)	0.140	1.62 (0.85, 3.08)	0.141
No	72 (47.1)	37 (53.6)	35 (41.7)		1.00	
Smoke inside home						
Yes	74 (48.4)	26 (38.2)	48 (56.5)	0.025	2.10 (1.09, 4.02)	0.026
No	79 (51.6)	42 (61.8)	37 (24.2)		1.00	

* Variables with ≤5 cell sizes were not included in the logistic regression analysis. ^†^ *p*-values reported from the chi-squared test for measuring the association between outcome and independent factor. ^‡^ *p*-values reported from the binary logistic regression analysis results. Heart-related illnesses were created by combining variables: ever having heart problems and ever having atrial fibrillation. Respiratory illnesses were created by combining variables: ever having COPD or emphysema, ever having asthma, ever having chronic bronchitis, and ever having pneumonia.

**Table 4:** Estimates of odds ratios (95% confidence intervals) and *p*-values based on multivariable logistic regression of the prevalence of poor sleep efficiency.

Variables	Adjusted Odds Ratio (95% CI)	*p*-Value
Sex		
Male	0.90 (0.39, 2.10)	0.811
Female	1.00	
Age groups		
18–54 years	4.82 (1.38, 16.84)	0.014
55 years and over	1.00	
Taking any prescription medication on a regular basis		
Yes	0.54 (0.21, 1.37)	0.537
No	1.00	
Chronic pain		
Yes	3.49 (1.28, 9.52)	0.015
No	1.00	
Ever had high blood pressure		
Yes	4.32 (1.42, 13.20)	0.010
No	1.00	
Smoke inside home		
Yes	2.92 (1.29, 6.58)	0.010
No	1.00	

**Table 5:** Results of univariable and multivariable linear regression analyses of log-transformed sleep onset latency (N = 126).

Variables	Univariable Regression			Multivariable Regression		
	Unstandardized Coefficients	Std. Error	*p*-Value	Unstandardized Coefficients (Transformed Back to Original Scale)	Std. Error (Transformed Back to Original Scale)	*p*-Value
Sex						
Male	−0.011	0.076	0.882	−0.095 (0.803)	0.083 (1.210)	0.259
Female (ref)						
Age groups						
18–54 years	0.206	0.100	0.041	0.255 (1.798)	0.119 (1.315)	0.034
55 years and over (ref)						
Body Mass Index						
Obese	0.042	0.075	0.580			
Overweight or neither overweight nor obese (ref)						
Education						
High school not completed	0.171	0.077	0.028			
High school or above (ref)						
Money left over at the end of the month						
Not enough money	0.129	0.094	0.173			
Just enough money	0.074	0.121	0.545			
Some money (ref)						
Drink caffeinated beverages within two hours of going to bed						
Yes	−0.120	0.077	0.125			
No (ref)						
Smoking status						
Current smoker	0.120	0.084	0.153			
Ex or never smoker (ref)						
Never smoker						
Physical activity at least 3 days per week						
Yes	0.026	0.077	0.741			
No (ref)						
Taking any prescription medication on a regular basis						
Yes	−0.111	0.075	0.141	−0.075 (0.841)	0.090 (1.230)	0.405
No (ref)						
Sleep medication						
Yes	0.130	0.114	0.255			
No (ref)						
Traditional medication used for sleep						
Yes	0.262	0.247	0.291			
No (ref)						
Ever had anxiety						
Yes	−0.168	0.084	0.050	−0.188 (0.648)	0.088 (1.225)	0.034
No (ref)						
Chronic pain						
Yes	0.029	0.088	0.739	0.232 (1.706)	0.099 (1.256)	0.021
No (ref)						
Ever had high blood pressure						
Yes	0.061	0.092	0.510			
No (ref)						
Heart-related illness						
Yes	−0.283	0.118	0.018	−0.330 (0.468)	0.132 (1.355)	0.014
No (ref)						
Ever had stroke						
Yes	0.005	0.432	0.991			
No (ref)						
Ever had high cholesterol						
Yes	0.004	0.119	0.974			
No (ref)						
Ever had diabetes						
Yes	−0.206	0.105	0.052			
No (ref)						
Respiratory illness (COPD/EMP/Asthma/CB/PNEU)						
Yes	−0.035	0.088	0.694			
No (ref)						
Ever had reflux/heartburn						
Yes	−0.029	0.082	0.726			
No (ref)						
Depression						
Yes	−0.104	0.083	0.214			
No (ref)						
Ever sinus problems						
Yes	−0.108	0.131	0.412			
No (ref)						
Sleep Apnea						
Yes	−0.259	0.192	0.180			
No (ref)						
Ever had post-traumatic stress disorder						
Yes	−0.276	0.121	0.024			
No (ref)						
Wake up during the night due to terrifying dreams or nightmares or flashbacks to a traumatic event						
Yes	−0.032	0.075	0.674			
No (ref)						
Wake up and get out of bed during the night						
Never (ref)	ref					
1–2 times	−0.057	0.111	0.611			
3 or more times	−0.138	0.124	0.269			
Housing Conditions						
Crowding						
≤2 people/bedroom (ref)						
>2 people/bedroom	0.124	0.085	0.146			
Damage caused by dampness						
Yes	−0.125	0.075	0.096			
No (ref)						
Moldy or musty smell						
Yes	−0.113	0.074	0.133			
No (ref)						
Signs of mold in any living areas in your house						
Yes	0.051	0.076	0.507			
No (ref)						
Smoke inside home						
Yes	0.136	0.074	0.067	0.247 (1.766)	0.083 (1.210)	0.003
No (ref)						

Heart-related illnesses were created by combining variables: ever having heart problems and ever having atrial fibrillation. Respiratory illnesses were created by combining variables: ever having COPD or emphysema, ever having asthma, ever having chronic bronchitis, and ever having pneumonia. Note: ‘ref’ refers to the reference category of the categorical variable.

## Data Availability

The First Nations community owns and controls the data, and the data are released as per the research agreements with the communities and the OCAP principles (https://fnigc.ca/ocap-training/) (accessed on 10 November 2023). Requests for data access can be made to the Chief and Council of the community at reception@beardysband.com.

## Abbreviations

FNSHP	First Nations Sleep Health Project
BMI	Body mass index
AW	Actiwatch
WASO	Wake time after sleep onset
PSQI	Pittsburgh Sleep Quality Index
SPSS	Statistical Package for the Social Sciences
SD	Standard Deviation
OR	Odds ratio
CI	Confidence interval
